# Activity of the Ubiquitin-activating Enzyme Inhibitor TAK-243 in Adrenocortical Carcinoma Cell Lines, Patient-derived Organoids, and Murine Xenografts

**DOI:** 10.1158/2767-9764.CRC-24-0085

**Published:** 2024-03-19

**Authors:** Yasuhiro Arakawa, Ukhyun Jo, Suresh Kumar, Nai-Yun Sun, Fathi Elloumi, Anish Thomas, Nitin Roper, Diana Grace Varghese, Naoko Takebe, Xiaohu Zhang, Michele Ceribelli, David O. Holland, Erin Beck, Zina Itkin, Crystal McKnight, Kelli M. Wilson, Jameson Travers, Carleen Klumpp-Thomas, Craig J. Thomas, Chuong D. Hoang, Jonathan M. Hernandez, Jaydira Del Rivero, Yves Pommier

**Affiliations:** 1 Laboratory of Molecular Pharmacology and Developmental Therapeutics Branch, Center for Cancer Research, NCI, NIH, Bethesda, Maryland.; 2 National Center for Advancing Translational Sciences, NIH, Bethesda, Maryland.; 3 Thoracic Surgery Branch, Center for Cancer Research, NCI, NIH, Bethesda, Maryland.; 4 Surgical Oncology Program, Center for Cancer Research, NCI, NIH, Bethesda, Maryland.

## Abstract

Current treatment options for metastatic adrenocortical carcinoma (ACC) have limited efficacy, despite the common use of mitotane and cytotoxic agents. This study aimed to identify novel therapeutic options for ACC. An extensive drug screen was conducted to identify compounds with potential activity against ACC cell lines. We further investigated the mechanism of action of the identified compound, TAK-243, its synergistic effects with current ACC therapeutics, and its efficacy in ACC models including patient-derived organoids and mouse xenografts. TAK-243, a clinical ubiquitin-activating enzyme (UAE) inhibitor, showed potent activity in ACC cell lines. TAK-243 inhibited protein ubiquitination in ACC cells, leading to the accumulation of free ubiquitin, activation of the unfolded protein response, and induction of apoptosis. TAK-243 was found to be effluxed out of cells by MDR1, a drug efflux pump, and did not require Schlafen 11 (*SLFN11*) expression for its activity. Combination of TAK-243 with current ACC therapies (e.g., mitotane, etoposide, cisplatin) produced synergistic or additive effects. In addition, TAK-243 was highly synergistic with BCL2 inhibitors (Navitoclax and Venetoclax) in preclinical ACC models including patient-derived organoids. The tumor suppressive effects of TAK-243 and its synergistic effects with Venetoclax were further confirmed in a mouse xenograft model. These findings provide preclinical evidence to support the initiation of a clinical trial of TAK-243 in patients with advanced-stage ACC. TAK-243 is a promising potential treatment option for ACC, either as monotherapy or in combination with existing therapies or BCL2 inhibitors.

**Significance:**

ACC is a rare endocrine cancer with poor prognosis and limited therapeutic options. We report that TAK-243 is active alone and in combination with currently used therapies and with BCL2 and mTOR inhibitors in ACC preclinical models. Our results suggest implementation of TAK-243 in clinical trials for patients with advanced and metastatic ACC.

## Introduction

Adrenocortical carcinoma (ACC) is a rare endocrine cancer with an annual incidence of 1.5 to 2 cases per million people (1, 2). In the United States, an estimated 300 people are diagnosed each year. If detected early, ACC can often be successfully treated surgically. However, nearly 70% of patients present with advanced ACC at the time of diagnosis, leading to a grim prognosis with an overall 5-year mortality rate of 75% to 90% and an average survival from the time of diagnosis of 14.5 months (3). The rarity of ACC makes it difficult to conduct large-scale clinical trials and discover novel therapies.

Mitotane (1,1 dichloro-2-(o-chlorophenyl)-2-(p-chloro-phenyl) ethane; o,p’-DDD) is the only FDA-approved treatment for advanced ACC. It was introduced more than 60 years ago and approved by the FDA in 1970 for metastatic ACC and adjuvant setting. In addition to mitotane, cytotoxic chemotherapy drugs, such as etoposide, doxorubicin, and cisplatin, are routinely used in combination (EDP-M) for unresectable or metastatic ACC as per the First International Randomized Trial in Locally Advanced and Metastatic Adrenocortical Carcinoma Treatment (FIRM-ACT) trial (4). However, the prognosis remains poor with a median overall survival of approximately 15 months even in the EDP-M treatment arm (4). Therefore, more effective therapies are needed for advanced ACC. Because of the rarity of the disease, preclinical models for ACC are limited with only few cell lines available (5, 6), which have not been included in the large drug screening efforts at the NCI, Broad-MIT or Sanger Institutes (reviewed in ref. 7).

The mechanism of action of mitotane remains poorly understood. In addition to downregulating steroid biogenesis by inhibiting sterol-O-acyl-transferase (SOAT1), mitotane induces lipid-induced endoplasmic reticulum (ER) stress and mitochondrial dysfunctions leading to apoptosis (8, 9). ER stress is typically caused by an abnormal accumulation of proteins in the ER, and the ubiquitin-proteasome system (UPS) is a key cellular mechanism that maintains intracellular protein homeostasis by degrading misfolded or damaged proteins. Although the unfolded protein response (UPR) is the main pathway of ER stress activation, impairment of lipid homeostasis, especially accumulation of free cholesterol, is known to activate this pathway (8, 10, 11). Kroiss and colleagues demonstrated that mitotane induces ER stress through lipotoxicity in ACC cell lines and synergizes with proteasome inhibitors, resulting in proteotoxic stress (12).

UPS not only maintains cellular protein homeostasis, but it also regulates signal transduction pathways, cell cycle progression, DNA damage repair, and apoptosis (13, 14). Thus, UPS is potentially associated with the pathogenesis of various types of cancers (13, 15, 16). UPS consists of two steps, protein ubiquitylation and degradation, with ubiquitylation taking place by the sequential action of ubiquitin-activating enzymes (E1), ubiquitin-binding enzymes (E2), and ubiquitin ligases (E3; ref. 14). UBA1 and UBA6 are ubiquitin-activating E1 enzymes that are the starting point of the ubiquitylation cascade; and UBA1 is the predominant E1 enzyme in humans.

Inhibition of E1 enzymes is a rational strategy for cancer therapy because it collectively inactivates unregulated pathologic pathways in cancer. TAK-243 is a first-in-class E1 inhibitor that binds to free ubiquitin and forms a TAK-243-ubiquitin adduct blocking the activation of E1 enzymes (17). As a result, TAK-243 inhibits intracellular ubiquitin binding, causing proteotoxic stress and ultimately killing cancer cells (17). To date, TAK-243 has been shown to exhibit antiproliferative activity in various human cancer models, including blood cancers (acute myeloid leukemia, B-cell lymphoma, multiple myeloma) and solid tumors (pancreatic cancer, small cell lung cancer, glioblastoma); it is in phase I clinical trials (18–23).

In this study, in collaboration with the National Center for Advancing Translational Sciences (NCATS), we screened the Mechanism Interrogation PlatEs (MIPE 5.0) compound library comprising 2,480 approved and investigational chemotherapeutic agents, as well as common medications for non-cancer indications drugs (24, 25) against three ACC cell lines (CU-ACC1, CU-ACC2, NCI-H295R). The screening identified several drugs targeting the UPS, suggesting that UPS is a valid therapeutic target for ACC. Among them, TAK-243 was identified as one of the most potent drugs.

## Materials and Methods

### Cell Culture and Reagents

NCI-H295R (RRID: CVCL_0458) and SW-13 (RRID: CVCL_0542) were purchased from ATCC. CU-ACC1 (RRID: CVCL_RQ00) and CU-ACC2 (RRID: CVCL_RQ01) were provided by Dr. K. Kiseljak-Vassiliades (5). NCI-H295R cells were grown in 1:1 DMEM:F12 (Thermo Fisher Scientific) supplemented with 2.5% Nu-Serum (Corning), 1% ITS media supplement (R&D Systems), and 1% Penicillin–Streptomycin (Thermo Fisher Scientific). SW13 was grown in DMEM (Thermo Fisher Scientific) supplemented with 10% FBS (GeminiBio) and 1% Penicillin–Streptomycin (Gibco). CU-ACC1 and CU-ACC2 ACC cell lines were grown in F medium [3:1 (v/v) Ham's F-12 Nutrient Mixture–DMEM (Thermo Fisher Scientific) supplemented with 5% FBS, 0.4 µg/mL hydrocortisone (Sigma-Aldrich), 5 µg/mL insulin (Sigma-Aldrich), 8.4 ng/mL cholera toxin (Sigma-Aldrich), 10 ng/mL EGF (Invitrogen), 24 µg/mL adenine (Sigma-Aldrich)] (5). All cell lines were cultured at 37°C in a humidified incubator with 5% CO_2_. Cell line identification tests were performed by short tandem repeat polymorphism analysis, using previously reported standard methods (5, 26). All cell lines were tested by MycoAlert *Mycoplasma* Detection Kit (Lonza) and confirmed to be *Mycoplasma* negative.

### Quantitative High-throughput Screening Using the MIPE 5.0 Library and Matrix Combination Screening

NCI-H295R, CU-ACC1, and CU-ACC2 cells were seeded into 1,536-well tissue culture-treated plates at a density of 500–1,000 cells/well in 5 µL growth medium using a Multidrop Combi dispenser. After cell addition, 23 nL of MIPE 5.0 compound (24, 25) was added to individual wells (11 concentrations were administered for all compounds in separate wells). A total of 3 µL of CellTiter-Glo (Promega) was loaded to each well and the plate was covered with a stainless steel lid and incubated at room temperature for 15 minutes. Luminescence was read using a Viewlux (PerkinElmer). Concentration–response curves for compounds were normalized against DMSO and empty well controls for each plate. All single-drug screening data are in Supplementary Table S1.

To test drug synergism, we performed a 10 × 10 matrix combination screening using 107 different drugs (all of which are in Supplementary Table S2). A 9-point concentration range with a 1:2 dilution between points was used for each drug, and cell viability was measured as in the single-drug screen (27).

### Cell Viability Assays

Three hundred cells were plated on 384-well white plates (Corning); after 24 hours of incubation, the indicated drugs were added, and cells were incubated for 72 hours. Cell viability was evaluated using CellTiter-Glo (Promega) with SpectraMax i3x (Molecular Devices) according to the manufacturer's instructions. For the MTT (3-[4,5-dimethylthiazol-2-yl]-2,5 diphenyl tetrazolium bromide) assay, 1 × 10^4^/well of H295R cells were seeded in 96-well plates and the indicated drugs were added after 24 hours and cultured for an additional 72 hours. Cell viability was measured using the TACS MTT Cell Proliferation Assay Kit (Trevigen). Drugs were obtained from the following sources: TAK-243, SJG-136, topotecan, doxorubicin, cisplatin, etoposide, and temozolomide were from Developmental Therapeutics Program (DTP, NCI); BAY-1895344, aldoxorubicin, entinostat, BMS-754807, brigatinib, ceritinib, Infigratinib, ulixertinib, berzosertib, navitoclax, capivasertib, MK-2206, XL-228, ribociclib, palbociclib, IPI-549, H-89, LY-2090314, cabozantinib, and everolimus from NCATS; bortezomib (5.04314) and mitotane (SML1885) from Sigma-Aldrich; OTS-167 (S7159) and pevonedistat (S7109) from Selleckchem.

### Patient-derived Organoid, Patient-derived Xenograft–derived Organoid Culture and Drug Treatment

ACC patient-derived organoids (PDO) were prepared from surgical specimens with the patient's written informed consent and ethical approval by the NIH Institutional Review Board. The study was also conducted in accordance with the Declaration of Helsinki. Some of the surgical specimens were immediately stored on ice in storage medium (1x DMEM/F12, 1x Glutamax, and 10 mmol/L HEPES buffer) for enzymatic degradation and organoid preparation. Each PDO was cultured in drops of Matrigel Growth Factor Reduced Basement Membrane Extract (Corning) and the medium was refreshed every 4 days. The culture medium was Advanced DMDM/F12 (Thermo Fisher Scientific) supplemented with 1x Glutamax (Gibco), 1x N2 supplement (Thermo Fisher Scientific), 1x B27 supplement (Thermo Fisher Scientific), 50 ng/mL EGF (Thermo Fisher Scientific), 20 ng/mL bFGF (STEMCELL Technologies), 20 ng/mL IGF-2 (STEMCELL Technologies), and 10 µmol/L Y-27632 (STEMCELL Technologies).

To create organoids from patient-derived xenografts (PDX), two ACC PDXs (#592788, #164165) were obtained from the NCI Patient-Derived Model Repository (NCI-PDMR; https://pdmr.cancer.gov/). These PDXs were first implanted subcutaneously in NSG mice for growth, and PDX-derived organoids (PDXO) were generated and maintained by the same process as PDO with minor modifications, the PDX tissues were magnetically labeled with a cocktail of mAb conjugated with MACS microbead to eliminate mouse cells using mouse cell depletion kit (Miltenyi Biotec).

For drug sensitivity studies, 4 × 10^5^ cells/mL were suspended in cold medium and mixed with an equal volume of Matrigel. A total of 10 µL of the organoid/Matrigel complex was dispensed into prewarmed 384-well opaque plates (2,000 cells/well). Twenty µL of medium was added on top of the organoids after Matrigel solidification. Forty-eight hours after the start of PDO culture, 2 × concentration of the drug in 30 µL medium was added in triplicate and the corresponding vehicle (DMSO) was used as control; after 72 hours of incubation, 20 µL of CellTiter-Glow (Promega) was added to each well. After incubation for 30 minutes at room temperature on a shaker, luminescence was measured with a SpectroMax i3x (Molecular Device). Supplementary Table S3 shows the clinical information for each PDO.

### Western Blot Analysis

Cells were lysed in RIPA buffer [150 mmol/L NaCl, 50 mmol/L Tris-HCl (pH 7.5), 1 mmol/L ethylenediaminetetraacetic acid (EDTA), 1% NP40, 0.1% SDS and 0.5% sodium deoxycholate, protease inhibitor cocktail (Cell Signaling Technology) and phosphatase inhibitor (Thermo Scientific)]. Cell lysates were loaded into wells of Novex Tris-Glycine gels (Invitrogen), electrophoresed, and transferred to Immun-Blot polyvinylidene difluoride membranes (Bio-Rad). Membranes were incubated with primary antibodies [SLFN11 (sc-515071, Santa Cruz Biotechnology), MDR-1 (C219, a gift from Dr. Robert W Robby at NCI), BCRP (BXP-21, a gift from Dr. Robert W Robby at NCI), Ubiquitin (#3933, Cell Signaling Technology), Ubiquityl-H2B (K120; #5546, Cell Signaling Technology), UBE1 (#MA5-32402, Thermo Fisher Scientific), PERK (sc-377400, Santa Cruz Biotechnology), Phospho-eIF2α (S51; #9721, Cell Signaling Technology), ATF-4 (#11815, Cell Signaling Technology), Phospho-IRE1 (ab124945, Abcam), ATF-6 (#65880, Cell Signaling Technology), Ubiquityl-PCNA (proliferating cell nuclear antigen; K164; #13439, Cell Signaling Technology), PARP (#9542, Cell Signaling Technology), cleaved caspase-3 (#9661, Cell Signaling Technology), GAPDH (GTX100118, GeneTex)] overnight in PBS-T buffer at 4°C, followed by incubation with horseradish peroxidase–labeled secondary antibodies (Cell Signaling Technology). Membranes were developed with Supersignal West Pico Plus or Femto Substrate (Thermo Fisher Scientific) according to the manufacturer's instructions and imaged with a ChemiDoc imaging system (Bio-Rad). Band intensities were quantified using ImageJ software (RRID:SCR_003070).

### DNA Synthesis Using 5-ethynyl-20-deoxyuridine Incorporation Assay and Flow Cytometry

Cells were seeded in 6-well plates, treated with the drug, and incubated with 10 mmol/L of 5-ethynyl-20-deoxyuridine (EdU) for 30 minutes before cell collection. EdU uptake was measured using the Click-iT Plus EdU Alexa Fluor 647 Flow Cytometry Assay Kit (Invitrogen) according to the manufacturer's protocol. Flow cytometry was performed by FACS Canto (Becton Dickinson), and results were analyzed by FlowJo software (RRID:SCR_008520).

### Animal Experiments

This study and animal usage were approved by the full board review of the NCI-Bethesda Animal Care and Use Committee (NCI-Bethesda Animal Study Proposal DTB-007). A total of 4 × 10^6^ H295R cells were suspended in 100 µL of Matrigel and inoculated subcutaneously into the right flank of NSG female mice. One week later, mice were divided into three groups (vehicle, TAK-243 10 mg/kg, TAK-243 20 mg/kg intraperitoneally twice weekly; *n* = 5 per group). A total of 4 × 10^6^ CU-ACC1 cells were inoculated subcutaneously into the right flank of NSG female mice as well. One week later, mice were divided into four groups (vehicle, TAK-243 10 mg/kg intraperitoneally twice a week, Venetoclax 100 mg/kg orally five times a week, TAK-243 + Venetoclax; *n* = 5 per group). Xenograft diameters and mouse weights were measured weekly, and on day 29 all mice were euthanized, and xenografts were harvested.

### IHC

Tumors were harvested on day 29 of the mouse xenograft experiment and IHC was performed using anti-multi-ubiquitin antibody (D058-3, MBL) and anti-cleaved caspase-3 antibody (#9661S, Cell Signaling Technology). Paraffin-embedded sections were heat-treated (95°C, 20 minutes) for antigen activation, then reacted with primary antibody at 300x dilution at 4°C overnight, followed by detection using the polymer method.

### Quantification and Statistical Analysis

Statistical analyses were performed using GraphPad Prism 9.0 software, and two-way ANOVA was used for *in vivo* time course data. Comparisons between groups were made using Tukey multiple comparisons test to calculate adjusted *P* values. Concentration–response curves for each drug were plotted and IC_50_ values were calculated using GraphPad Prism 9.0 software. Drug synergism by combination index (CI) was calculated using CompuSyn software (https://www.combosyn.com/). A CI < 1 was considered synergistic, especially < 0.5 was considered strongly synergistic.

### Data Availability

All drug screening data obtained in this study are available in Supplementary Tables S1 and S2. All other data in this article can be obtained from the corresponding author upon reasonable request.

## Results

### High-throughput Screening Uncovers TAK-243 as a Potent Agent Against ACC Cell Lines

To identify compounds active against ACC, the MIPE 5.0 library developed by the NCATS was screened in three ACC cell lines (CU-ACC1, CU-ACC2, and NCI-H295R). The library contains 2,480 approved or investigational drugs based on mechanisms focused on cancer therapy, with a mechanistic diversity of more than 800 different molecular targets, while preserving redundancy by including multiple inhibitors of well-explored targets (24, 25).

**FIGURE 1:**
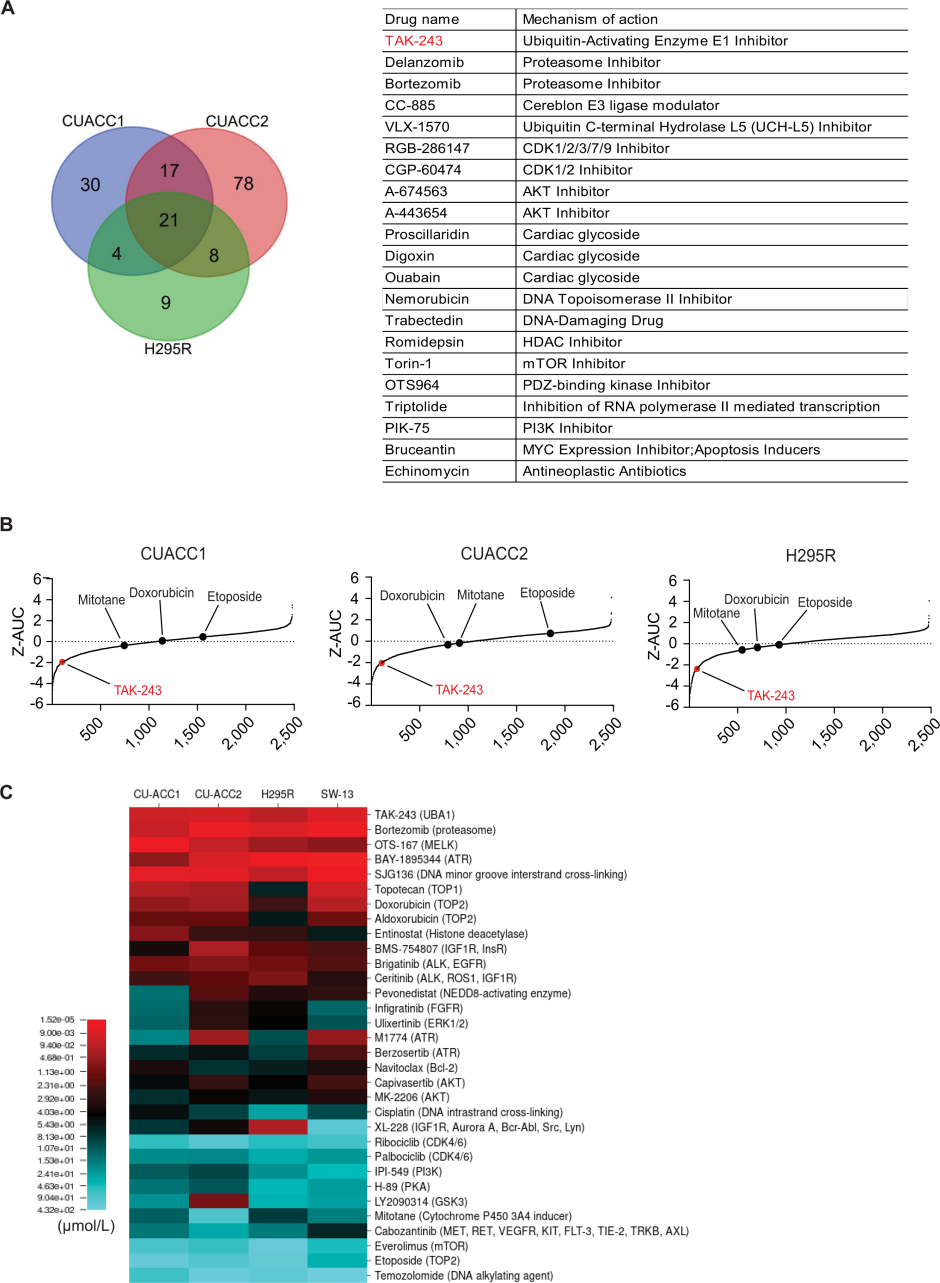
NCATS screening identifies TAK-243 as one of the most potent drugs against ACC cell lines. **A,** CU-ACC1, CU-ACC2, and NCI-H295R cell lines were screened with the NCATS MIPE v5.0 (MIPE 5.0) library of approved and investigational drugs (*n* = 2,480). Drugs with Z-AUC values less than −1.5 and good-quality dose–response curves were selected. The number of drugs effective in the three cell lines is shown in the Venn diagram (left). The 21 drugs commonly effective in the three cell lines and their mechanism of action were tabulated. **B,** Drugs were arranged in Z-AUC order for the three cell lines tested, with red dots indicating TAK-243. The positions of mitotane, doxorubicin, and etoposide were also shown. **C,** Heat map of IC_50_ values of selected drugs with highest and lowest activity against the three ACC cell lines and SW-13 (small cell carcinoma cell line of adrenocortical origin). Drug targets were listed in parentheses.

We used AUC for a comprehensive comparison of drugs. We also calculated AC_50_ values (concentration at which 50% of the maximum activity is observed) for the drugs, but due to the shape of the concentration–response curves, this approach did not yield results for many drugs (Supplementary Table S1). Thus, we analyzed the data by Z-transformed AUC in all three cell lines and selected drugs with Z-AUCs less than −1.5 and highest quality dose–response curves (curve classes −1.1, −1.2, −2.1, −2.2; ref. 28). The mean Z-AUC score of each drug in each of the three cell lines was calculated and drugs were ranked on the basis of their activity; 326 drugs showed significant activity with a mean Z-AUC scores < −1 and pathway enrichment (Supplementary Table S1). Drugs targeting the proteasome 26S subunit PSMD1 (seven drugs), CDK1 (11 drugs), HDAC1 (10 drugs), and IGFR1 (four drugs) had high hit rates. Thirty-two drugs targeting the UPS were included in MIPE 5.0, and 14 drugs (hit rate 43.8%) were found to be active in ACC (Fig. 1A and B; Supplementary Table S1). In addition, many drugs targeting the PI3K-AKT-mTOR pathway were found active, including PIK3CA (10 drugs), AKT1 (five drugs), and mTOR (six drugs; Fig. 1A and B; Supplementary Table S1). Thus, among the 21 drugs found effective across the three cell lines, we found five targeting the UPS (Fig. 1A and B).

To validate our screening results, we tested the molecularly targeted drugs found in our NCATS screen and anticancer drugs used clinically in the treatment of ACC (topoisomerase inhibitors, platinum, mitotane) in our three ACC cell lines and the SW-13 cell line (a small cell carcinoma cell line of adrenocortical origin; Fig. 1C). Most notably, drugs targeting the UPS, such as TAK-243 and bortezomib, were highly potent in the ACC cell lines (Fig. 1C).

### TAK-243 is Cytotoxic at Nanomolar Concentrations in ACC Cell Lines

**FIGURE 2:**
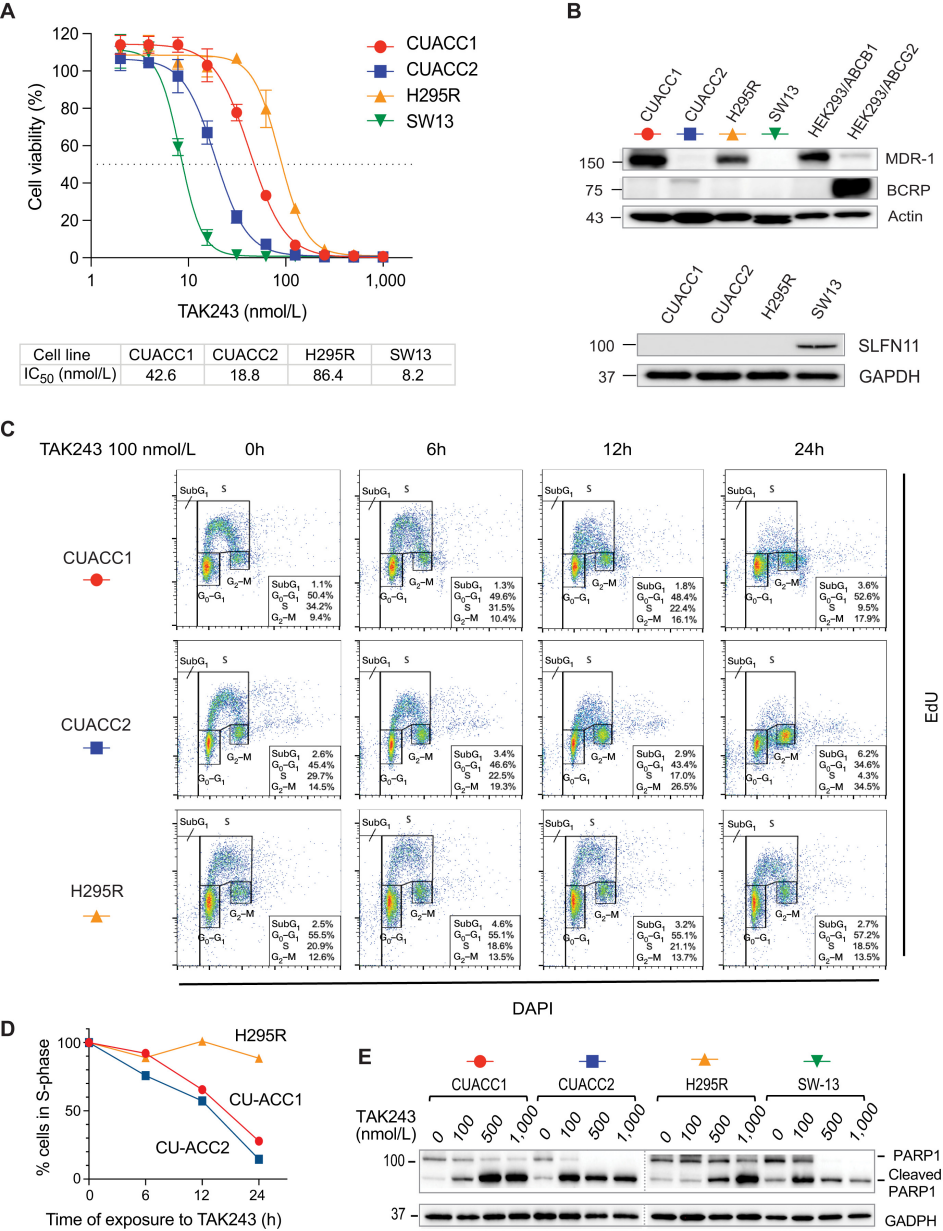
Cytotoxicity of TAK-243 on ACC cell lines. **A,** Concentration–response curve and IC_50_ values of TAK-243 in ACC cell lines and SW-13. Cell viability after 72 hours under the indicated drug concentrations was measured by the CellTiter-Glo viability assay. **B,** Expression of ABC transporters (MDR-1, BCRP) and SLFN11 in ACC cell lines and SW-13. Proteins were extracted from each cell line and expression of SLFN11, MDR-1, and BCRP were evaluated by Western blotting. **C,** Effects of TAK-243 on DNA synthesis and cell cycle. CU-ACC1, CU-ACC2, and NCI-H295R cells were treated with 100 nmol/L TAK-243 for the indicated times and pulse-labeled with EdU (10 µmol/L) for 30 minutes before harvest. Cellular EdU uptake and cell cycle were analyzed by flow cytometry. **D,** Change in percentage of S-phase after TAK-243 treatment. **E,** ACC cell lines and SW-13 were treated with TAK-243 at the indicated concentrations for 24 hours, and PARP cleavage was detected by Western blotting.

Consistent with the NCATS and our own drug screening results (Fig. 1), TAK-243 showed nanomolar activity against in the ACC cell lines with IC_50_ values in the order CU-ACC2 < CU-ACC1 < NCI-H295R (Fig. 2A). Bortezomib, a clinical drug targeting the UPS by inhibiting the proteasome in myeloma and mantle cell lymphomas, also exhibited IC_50_ at the nanomolar level in ACC cell lines, but a notable fraction of cells survived at high concentrations in CU-ACC1 and H295R (Supplementary Fig. S1A), which led us to pursue our studies withTAK-243.

Because TAK-243 has been reported to be a substrate for the drug efflux P-glycoprotein pumps (29), we tested the expression of the ATP-binding cassette transporters MDR-1 (*ABCB1*) and BCRP (*ABCG2*). Consistent with the report that genetically engineered ABCB1-overexpressing colon SW620, kidney HEK, and mouth epithelial KB cells are highly resistant to TAK-243, we found that the NCI-H295R and CU-ACC1 cell line overexpress MDR1 and are the most resistant to TAK-243 (Fig. 2A and B). This result was confirmed by RNA sequencing (RNA-seq), which showed high ABCB1 expression in the ACC1 and NCI-H295R ACC cell lines (Supplementary Fig. S1B).

As it has been recently reported that cells with inactivated Schlafen 11 (SLFN11) are selectively sensitive to TAK-243 (30), we evaluated the expression of SLFN11 by Western blotting and found that none of the ACC cell lines expressed SLFN11 (Fig. 2B, bottom). This result was confirmed by RNA-seq, and DNA methylation array (850k Epic array), which showed promoter hypermethylation in the three ACC cell lines (Supplementary Fig. S1). These results demonstrate that lack of SLFN11 in the three ACC cell lines is epigenetically driven and associated with sensitivity of the ACC cell lines to TAK-243 (30).

We also examined changes in DNA replication and cell cycle after TAK-243 treatment (30) after pulse incorporation of the thymidine analog EdU and double staining with DAPI by flow cytometry (Fig. 2C). Reduction of EdU uptake and accumulation of cells in G_2_–M were higher in the most sensitive CU-ACC1 and CU-ACC2 cell lines than the resistant NCI-H295R cells (Fig. 2C and D).

To determine whether TAK-243 induces apoptosis in the ACC cell lines we measured PARP1 cleavage. We used a wide concentration range of TAK243 from 100 to 1,000 nmol/L to detect PARP cleavage at 24 hours posttreatment. CU-ACC2 and CU-ACC1 cells showed apoptosis upon treatment with TAK-243 at 100 nmol/L for 24 hours (Fig. 2E), while in NCI-H295R cells, PARP cleavage was observed at > 500 nmol/L of TAK-243. Thus, CU-ACC2 cells, which express neither the MDR1 drug efflux transporter nor SLFN11, show a marked decrease in DNA replication and G_2_–M-phase arrest after TAK-243 treatment, while being the most sensitive ACC cell line to TAK-243. By contrast, NCI-H295R cells, which overexpress MDR1 are relatively resistant to TAK-243; although still susceptible to TAK-243 with an IC_50_ of 86 nmol/L.

### TAK-243 is Cytotoxic at Nanomolar Levels in ACC PDOs

**FIGURE 3:**
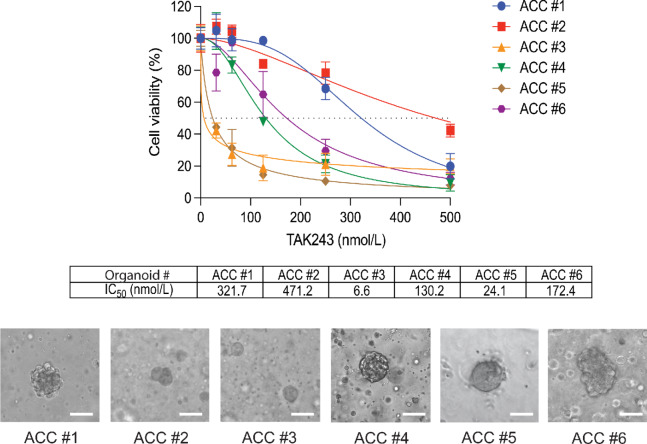
Activity of TAK-243 on ACC PDOs. Concentration–response curves and IC_50_ values of TAK-243 for ACC PDOs. The indicated concentrations of TAK-243 were administered to organoids derived from patients with ACC for 72 hours and cell viability was determined. Representative brightfield images of organoids were also shown (20x magnification, scale bar 50 µm).

Because organoid models reproduce the genetic and morphologic heterogeneity of cancer tissues (31), we tested the cytotoxicity of TAK-243 using three-dimensional (3D) organoids prepared from surgical specimens from patients with ACC treated at the NCI. We generated six ACC organoids (ACC #1–6) derived from 5 patients and determined their response to TAK-243 (Fig. 3). Clinical information for each organoid is summarized in Supplementary Table S2. IC_50_ values in the organoids were all in the nanomolar range, suggesting that TAK-243 is effective in organoids derived from patients with ACC as it in ACC cell lines.

### TAK-243 Rapidly Depletes Ubiquitylated Proteins, Causing UPR

**FIGURE 4:**
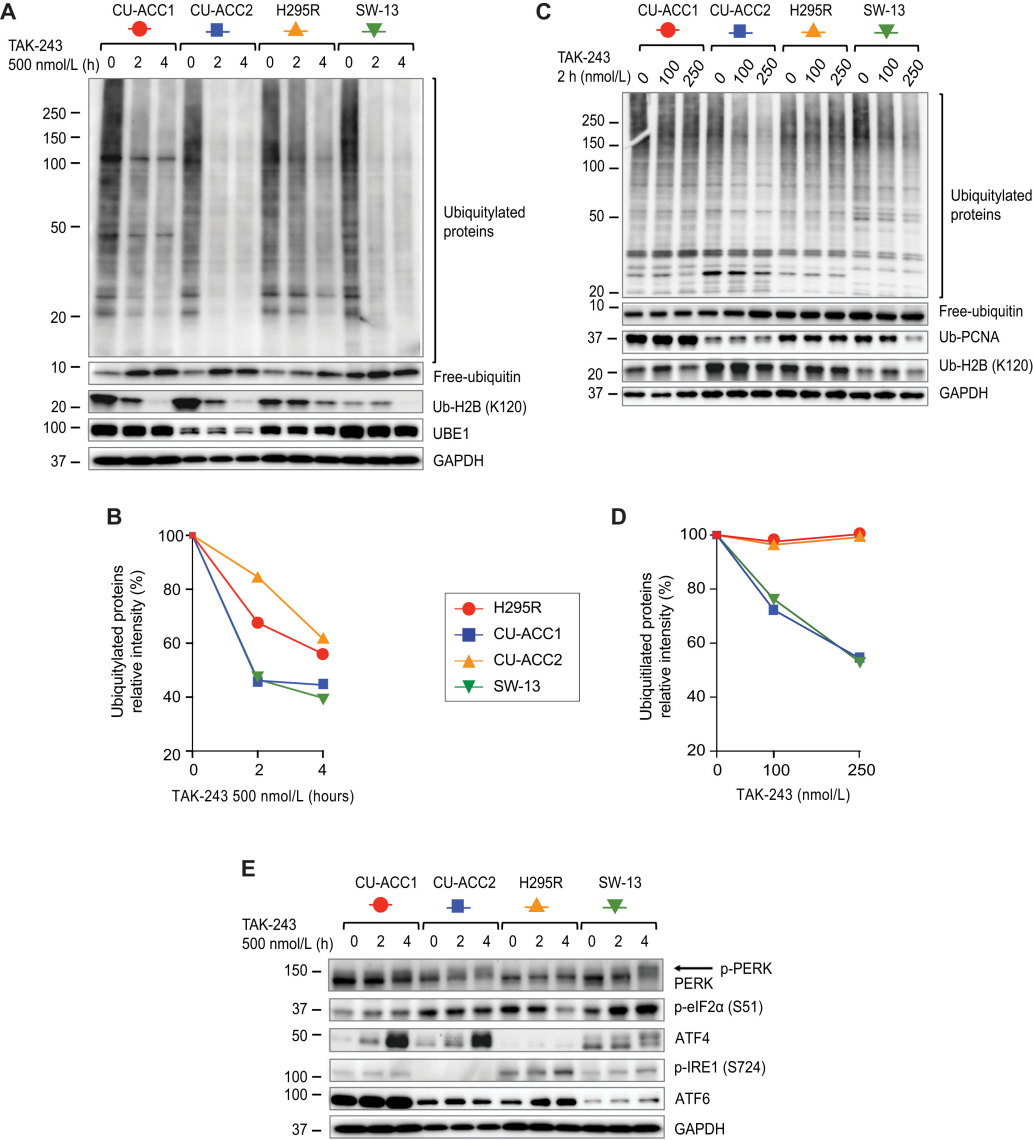
TAK-243 inhibits ubiquitylation and induces UPR in ACC cell lines. **A,** Time course for ubiquitylated proteins and free ubiquitin in cells treated with TAK-243 (500 nmol/L, 2 or 4 hours). The indicated proteins were evaluated by Western blotting. **B,** Quantitation of the data shown in A. **C,** Concentration response of ubiquitylated proteins and free ubiquitin in cells treated with the indicated concentrations of TAK-243 for 2 hours. The indicated proteins were evaluated by Western blotting. **D,** Quantitation of the data shown in B. **E,** UPR induced by TAK-243 (500 nmol/L, 2 or 4 hours). The samples were from the same cellular lysates as in A, and GAPDH blots were intentionally reused for presentation. The indicated proteins were evaluated by Western blotting.

As TAK-243 is an established ubiquitin-activating enzyme inhibitor that has never been tested in ACC cell lines (17), we examined its effects on protein ubiquitylation and free ubiquitin. The three ACC cell lines and SW-13 were treated with 500 nmol/L TAK-243, and changes in ubiquitylated protein and UPR were evaluated over time (Fig. 4A). Ubiquitylated proteins decreased rapidly in CUACC-2 and SW-13 after TAK-243 treatment, whereas the decrease was slower in CUACC-1 and NCI-H295R. The rapid decrease in ubiquitylated proteins correlates with the sensitivity of the cell lines to TAK-243 (Figs. 2 and 4). Ubiquitylated histone H2B also decreased rapidly after TAK-243 treatment in all cell lines, and less rapidly in the most resistant NCI-H295R cell line (Fig. 4A and B). In parallel, TAK-243 increased free ubiquitin (Fig. 4A).

Even when TAK-243 was used at low concentrations (100, 250 nmol/L) for 2 hours, ubiquitylated proteins were significantly reduced in CU-ACC1 and SW-13 (Fig. 4C and D). Ubiquitylated histone H2B and ubiquitylated PCNA also decreased, but the overall decrease in ubiquitylated proteins was the most obvious pharmacodynamic biomarker demonstrating that TAK-243 effectively inhibits ubiquitylation in ACC, as consequence of its established E1 inhibitory activity.

Inhibition of ubiquitylation by TAK-243 has been shown to cause strong UPR due to the accumulation of unfolded and misfolded proteins (20, 22). Testing the three UPR pathways (PERK-eIF2α-ATF4, IRE1-XBP1, and ATF6) showed that the PERK pathway was rapidly activated in CU-ACC1, CU-ACC2, and SW-13 after TAK-243 treatment. In contrast, the IRE1 and ATF6 pathways were noticeably activated in NCI-H295R, indicating that the UPR pathways activated by TAK-243 differ among cell lines (Fig. 4E).

### Synergy Between TAK-243 and Mitotane and Additive Effects of TAK-243 with Etoposide/Cisplatin

**FIGURE 5:**
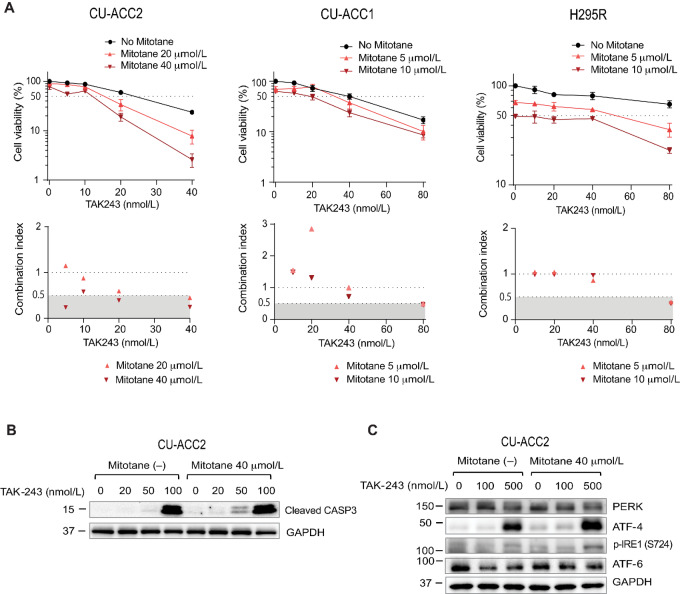
Synergy between TAK-243 and mitotane. **A,** Top: The indicated ACC cell lines were treated with TAK-243 with or without mitotane for 72 hours. Bottom: CI values were calculated and plotted. **B,** Enhanced induction of apoptosis by the TAK-243 + mitotane combination was measured as caspase-3 cleavage. **C,** Enhanced UPR induction by the TAK-243 + mitotane combination. CU-ACC2 cells were treated with or without mitotane (40 µmol/L) for 24 hours before adding the indicated concentrations of TAK-243 for 4 hours. UPR proteins were evaluated by Western blotting.

As TAK-243 is likely to be used clinically in combination, we first studied mitotane, the only FDA-approved and widely used treatment for advanced ACC. The IC_50_ values of mitotane against the ACC cell lines were above 10 µmol/L (vertical axis viability values in Fig. 5A), with CU-ACC2 being the most resistant and NCI-H295R the most sensitive to mitotane. Combining TAK-243 with mitotane showed synergistic or additive activity depending on the cell line. In CU-ACC2, TAK-243 was synergistic with mitotane with a CI < 0.5, which is considered to represent a highly synergistic effect (32). In CU-ACC1, the CI ranged with the concentration of TAK-243; in NCI-H295R, the two drugs were additive or synergistic, and the CI was around 1 at 40 nmol/L TAK-243 (reflecting an additive effect), and about 0.5 at 80 nmol/L TAK-243 (reflecting a synergistic effect).

We also compared apoptosis and UPR induction by TAK-243 in the presence of mitotane in the CU-ACC2 cell line with the strongest synergy between mitotane and TAK-243 (Fig. 5B and C). As assessed by cleaved caspase-3, apoptosis was induced at lower concentrations of TAK-243 when TAK-243 was combined with mitotane (Fig. 5B). Expression of ATF4 and p-IRE1 was also enhanced by the combination of mitotane with TAK-243 (Fig. 5C) Thus, the combination of TAK-243 and mitotane enhances UPR in ACC cell lines and can induce apoptosis at low concentrations of TAK-243.

Because the topoisomerase inhibitor etoposide and the cross-linking agent cisplatin are commonly used as standard first-line chemotherapy in advanced-stage ACC (4), we tested whether these drugs also showed a synergistic effect with TAK-243. However, only additive effects were observed without synergy in the combinations of TAK-243 with either etoposide or cisplatin (Supplementary Fig. S2).

### Identification of BCL2 and mTOR Inhibitors as Synergistic with TAK-243

**FIGURE 6:**
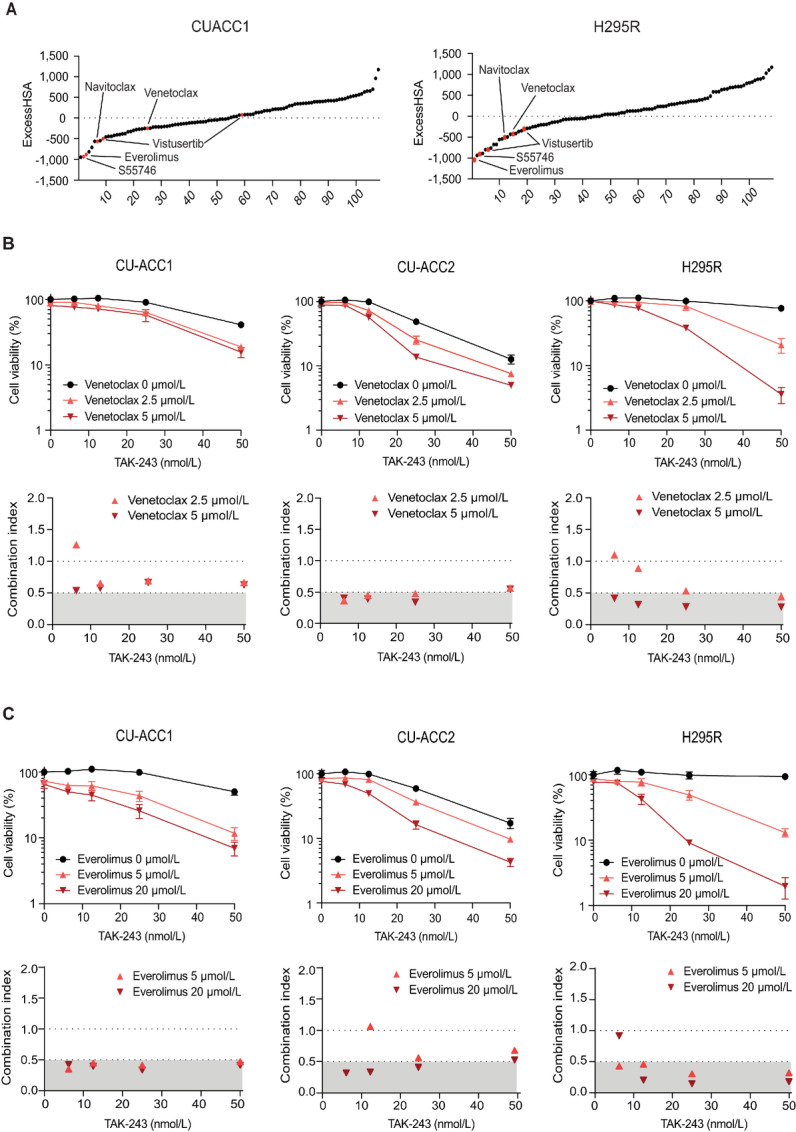
NCATS screening for synergistic drug combinations with TAK-243 in the CU-ACC1 and NCI-H295R cell lines. One hundred and seven drugs were tested in combination with TAK-243. **A,** Overall result of the screen with drugs listed in ExcessHSA order. The BCL2 inhibitors (S55746, Navitoclax and Venetoclax) and the mTOR inhibitors (Everolimus and Vistusertib) were indicated as red dots. **B** and **C**, Independent validation of the NCATS data for the BCL2 and mTOR inhibitors. ACC cell lines were treated with TAK-243 at the specified concentrations for 72 hours with or without Venetoclax (B) and Everolimus (C). Cell viability was assessed by CellTiter-Glo. Error bars represent SD of triplicates; CI values were also plotted.

To identify novel drug combinations synergistic with TAK-243 in the ACC cell lines CU-ACC1 and NCI-H295R, we performed a robotic screening of 107 different drugs including agents targeting the UPS, drugs related to ER stress, and agents targeting pathways that were effective in single-agent screening (Supplementary Table S3). When synergistic effects were assessed with ExcessHSA, the BCL2 inhibitors (Navitoclax, Venetoclax, S55746) and the mTOR inhibitors (Everolimus, Vistusertib) were top hits in both cell lines (Fig. 6A; Supplementary Table S3). We independently confirmed the synergistic effects of the BCL-2 inhibitors Venetoclax and Navitoclax and of the mTOR inhibitor Everolimus (Fig. 6B and C; Supplementary Fig. S3A) in the ACC cell lines. We further validated the synergistic effect of mitotane and Venetoclax with TAK-243 in H295R cells in MTT assays, a non–ATP-dependent measure of cell viability (Supplementary Fig. S3B).

**FIGURE 7:**
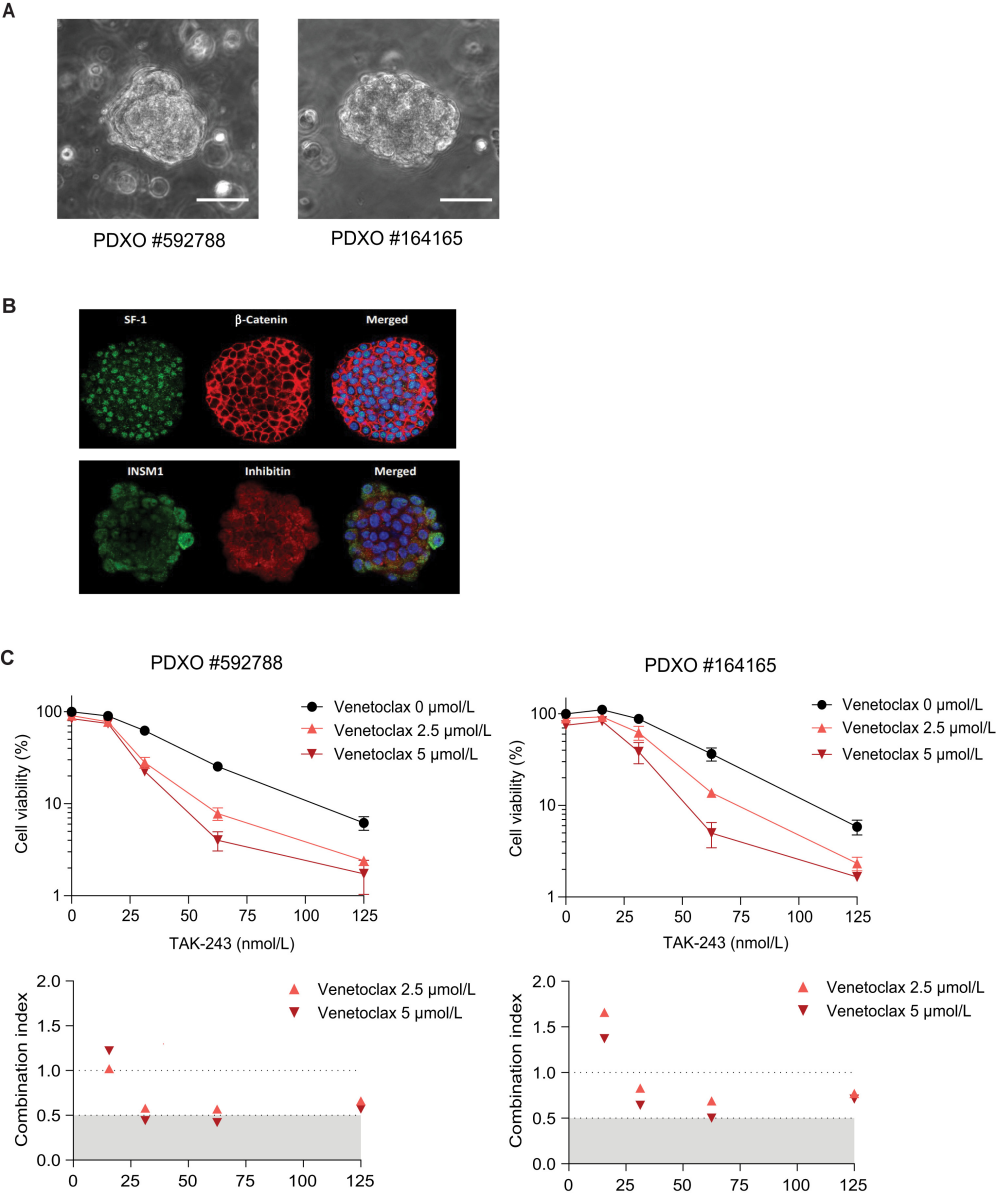
Synergy between TAK-243 and Venetoclax in organoids generated from patient-derived xenograft (PDXO). **A,** Representative brightfield images of PDXOs (20x magnification, scale bar 50 µm). **B,** Phenotypic characteristics of PDXOs. Expression of biomarkers routinely used to diagnose ACC, including inhibin-α, SF-1, INSM-1, and β-catenin in ACC-PDX-derived organoids using immunofluorescence assay. **C,** Top: The indicated ACC PDXOs were treated with TAK-243 with or without Venetoclax for 72 hours. Bottom: Plots of the corresponding CI values.

In addition, we tested two PDX models from NCI-PDMR, from which we were able to develop organoids (Fig. 7A). Immunostaining of the organoid (#592788) was positive for molecules characteristic of ACC, including SF-1, β-catenin, INSM-1, and inhibin-α (Fig. 7B; refs. 33, 34). The synergistic effect of TAK-243 and Venetoclax was recapitulated in these organoids (Fig. 7C) with IC_50_ values for TAK-243 as 38.2 nmol/L (PDXO#592788) and 46.3 nmol/L (PDXO#164165). Thus, we conclude that TAK-243 is effective both in ACC cell lines and against PDXO at nanomolar concentrations in combination with the BCL2 inhibitor Venetoclax.

### TAK-243 is Active and Synergistic with Venetoclax in Mouse Xenograft Models

**FIGURE 8:**
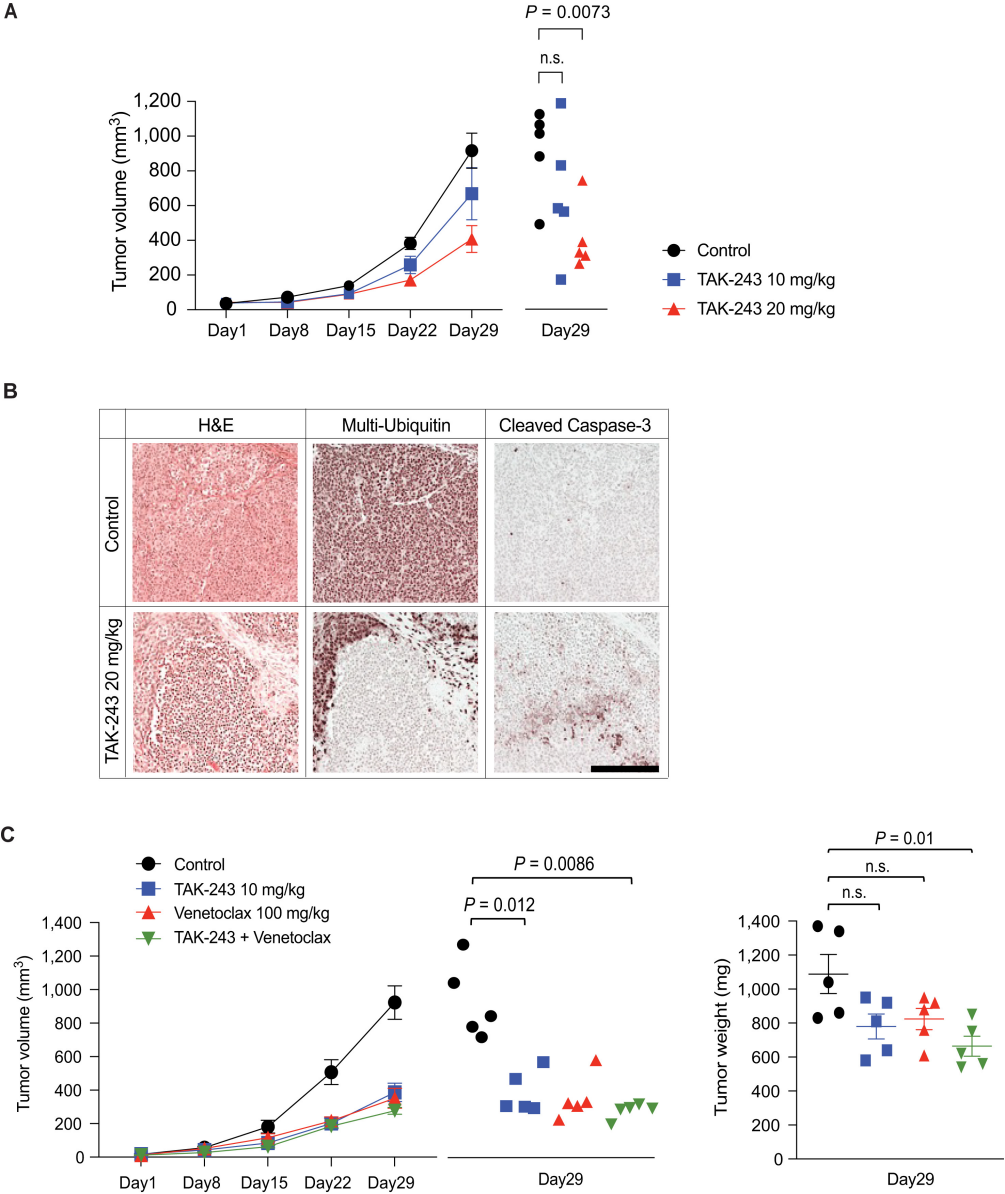
TAK-243 inhibits tumor growth in ACC xenograft models and synergizes with the BCL2 inhibitor Venetoclax. **A,** H295R xenografts were treated with TAK-243 (10 and 20 mg/kg) intraperitoneally twice weekly and mean tumor volume was evaluated. Error bars indicate SEM (*n* = 5); tumor volume for each mouse on day 29 was shown individually on the right. **B,** Xenograft tissues collected on day 29 (H&E staining, immunostaining for anti-multi-ubiquitin and anti-cleaved caspase-3 antibodies). Top panel: control, bottom panel: TAK-243 (20 mg/kg) group. Scale bar: 200 µm. **C,** Left: CU-ACC1 xenografts were treated with TAK-243 (10 mg/kg) intraperitoneally twice weekly, venetoclax (100 mg/kg) orally five times weekly, and a combination of TAK-243 and venetoclax, and mean tumor volume was evaluated. Error bars indicate SEM (*n* = 5); tumor volume of each mouse on day 29 was shown separately. Right: Tumor weight of each treatment group on day 29. Horizontal lines indicate mean values and error bars indicate SEM.

To investigate the *in vivo* activity of TAK-243, we first used the H295R mouse xenograft model, in which tumor growth was significantly inhibited in the TAK-243 20 mg/kg group compared with the control group (Fig. 8A). Pharmacodynamic studies showed that TAK-243 20 mg/kg treatment decreased ubiquitylated proteins in H295R xenografts and induced apoptosis as indicated by caspase-3 cleavage (Fig. 8B).

We also tested the CU-ACC1 xenograft model with TAK-243 alone and combination with Venetoclax. Tumor volume on day 29 was significantly smaller in the TAK-243 10 mg/kg group and in the TAK-243 plus Venetoclax group compared with the control group (Fig. 8C). Comparing tumor weights collected on day 29, only the TAK-243 and Venetoclax combination treatment group showed statistically significant tumor growth inhibition compared with the control group (Fig. 8C). IHC of the harvested xenografts also showed a strong induction of apoptosis as indicated by cleaved caspase-3 positivity in the combination therapy group (Supplementary Fig. S4A). No differences were observed in the weight changes of the mice in each group (Supplementary Fig. S4B and S4C) indicating that the combination treatments were effective and tolerated.

## Discussion

Because ACC is an orphan disease and no new drugs have been approved for more than five decades, we performed large scale drug screening in ACC preclinical models and identified TAK-243, a first-in-class ubiquitin-activating enzyme E1 inhibitor, as a potent agent active at nanomolar concentrations by itself and synergistic with the current treatments of ACC. We also report that TAK-243 is synergistic with the BCL2 inhibitor Venetoclax.

Our study extends previous drug screen efforts for ACC, which used the NCI-H295R, SW-13, and BD140A cell lines (35, 36). However, SW13 is not an unambiguous ACC cell line but a small cell cancer cell line of the adrenal gland (37), and the characteristics of BD140A cells are unknown (36). In our study, we also used the NCI-H295R, an established preclinical model of ACC, and two other ACC cell lines (CU-ACC1, CU-ACC2), whose cellular origin and characteristics have been reported in detail (5), to identify drugs that are commonly potent against them. NCI-H295R and CUACC1 are steroid-producing and have mutations in the β-catenin gene (*CTNNB1*), while CUACC2 is a steroid-nonproducing cell line established from a patient with Lynch syndrome (5). The Venn diagram of our drug testing (Fig. 1A) showed many drugs that were effective in only one or two cell lines, suggesting that it is important to ensure as much diversity as possible in preclinical models of ACC. Future work may be expanded to include a more diverse range of ACC cell lines and organoids.

Of the 2,480 drugs included in the NCATS MIPE 5.0 library, 21 were active across the three ACC cell lines (Fig. 1A). Among them, five target the UPS, including TAK-243. In addition, pathway analysis was performed to tabulate 326 drug targets with an average Z-AUC score of less than −1 across the three cell lines. Thirty-two UPS-targeted agents were tested in MIPE 5.0, of which 14 (43.8% hit rate) showed activity in the ACC cell lines. Drugs targeting CDK1, HDAC1, IGFR1, and the PI3K-AKT-mTOR pathway were also enriched (Supplementary Table S1). Proteasome inhibitors (12, 38), CDK inhibitors (38, 39), IGFR1 inhibitors (40, 41) and mTOR inhibitors (41, 42) have been reported to be effective in preclinical models of ACC, supporting the validity of our screen. It is therefore significant that we identified TAK-243, a novel and specific ubiquitin-activating enzyme E1 inhibitor (17) as one of the top hits. We confirmed that TAK-243 was effective in the ACC cell lines at nanomolar concentrations that are readily achievable in patients. Clinically used UPS-targeting drugs also include proteasome inhibitors, and although bortezomib had a low IC_50_ in our ACC cell lines, a significant fraction of CU-ACC1 and NCI-H295R cells were still viable at high bortezomib concentrations. On the basis of these findings, we focused on TAK-243.

As predicted from its selective targeting of the E1 ubiquitin-activating enzyme (17), TAK-243 decreased cellular ubiquitylation, leading to accumulation of free ubiquitin, which is followed by an UPR (Fig. 4). The early decrease in ubiquitylated proteins after TAK-243 treatment correlated with the cytotoxic activity of TAK-243. The reduction in ubiquitylated proteins could be seen early after drug administration and at low concentrations, making it a potential surrogate marker for predicting the activity of TAK-243. Following ER stress and UPR, we found that TAK-243 suppresses DNA replication in ACC cells with accumulation of cells in G_2_–M-phase, followed by apoptosis (Fig. 2).

Expression of MDR1 and SLFN11 are potential predictors of TAK-243 response. Increased expression of the ABC transporter MDR1 has been associated with decreased TAK-243 sensitivity (29). Accordingly, we found that the ACC cell lines, NCI-H295R and CU-ACC1, which express *ABCB1*, the gene encoding MDR1/PgP, are less sensitive to TAK-243 than the CU-ACC2 cells (Fig. 2A and B). This result is consistent with the fact that ACC is a known tumor with high MDR1 expression (43) (Supplementary Fig. S5). Yet even NCI-H295R and CU-ACC1 cells expressing MDR1, TAK-243 had IC_50_ values within nanomolar range, likely achievable in the clinic.

We recently reported that lack of *SLFN11* expression induces proteotoxic stress and increases the sensitivity of cancer cells to TAK-243 in isogenic leukemia and prostate cancer cell line models (30). The Cancer Genome Atlas (TCGA) data analysis and of our own patient data show that ACC is one of the tumors with the lowest *SLFN11* expression (Supplementary Fig. S5; ref. 44), which may explain the activity of TAK-243 compared with DNA-targeted genotoxic agents that are generally less active in the large fraction of cancers that do not express *SLFN11* (45).

The development of patient-derived 3D organoid models has advanced the field of cancer biology. However, 3D organoid models of endocrine cancers have rarely been reported and their application as a therapeutic model has not yet been realized (46). In this study, we have established PDO and PDXO from patients with ACC, which until now lacked preclinical models. Although our organoids could not be passaged for an extended period, we were able to perform drug sensitivity tests during the first two to three passages. In our PDO and PDXO from ACC, the IC_50_ values of TAK-243 were in the nanomolar range, which confirms that TAK-243 is effective in different ACC models.

Our results in the H295R and CU-ACC1 mouse xenograft models confirmed the activity of TAK-243 in a broad range of ACC preclinical models (Fig. 8A and C). In pharmacodynamic study of H259R xenograft, TAK-243 effectively inhibited protein ubiquitylation and induced apoptosis as indicated by cleaved caspase-3 (Fig. 8B). Furthermore, even doses of TAK-243, which clearly inhibited tumor growth, TAK-243 was well tolerated and mice showed no loss of body weight (Supplementary Fig. S4B and S4C). A previous study using a mouse xenograft model showed that the MTD of TAK-243 in SCID mice was 23–26 mg/kg twice weekly intravenously (17). Other studies using NSG mice showed no weight loss or abnormal blood biochemistry and no gross or histologic organ changes at 20 mg/kg twice weekly subcutaneously (18). Unfortunately, the clinical study of TAK-243 in patients with advanced malignant solid tumors (NCT02045095) was put on hold in the early dose-escalation phase due to the restructuring of the sponsor's pipeline program, the adverse effect profile of TAK-243 in humans still needs to be fully established.

Anticipating that TAK-243 would be used together with clinically approved anticancer agents, to seek synergistic combinations without overlapping toxicity we tested the combination of TAK-243 with the drugs used in the standard ACC chemotherapy regimens. We found more than an additive effect between TAK-243 and mitotane and additive effects of TAK-243 with etoposide and cisplatin, which justifies the potential use of TAK-243 in combination with the existing ACC regiments. By systematically screening drug combinations, we discovered that BCL2 and mTOR inhibitors are highly synergistic with TAK-243 (Fig. 6; Supplementary Table S1). Synergy between TAK-243 and Venetoclax was confirmed in all our ACC cell lines and PDXO models (Fig. 7). Mouse xenograft experiments also showed that TAK-243 is synergistic with Venetoclax (Fig. 8C; Supplementary Fig. S4A). Notably, ACC express high transcript levels of the proapoptotic *BAX* and low levels of *BCL2* (Supplementary Fig. S5B), suggesting that inhibition of BCL2 could shift the proapoptotic balance in ACC treated with Venetoclax. BCL2 inhibitors (Venetoclax, Navitoclax) are already used in clinically in other cancers and their side effect profiles are well understood, justifying them for planning future clinical trials in combination therapy.

Of note, in ACC patient samples from TCGA and our clinic, *UBA1* transcripts are consistently overexpressed (Supplementary Fig. S6), and upregulation of the E1 and E2 enzymes of the ubiquitin pathway is associated with Ki67 expression, a poor prognostic factor for ACC. As our study demonstrates the potential of TAK-243 as a new approach to treat ACC, it also raises fundamental questions regarding the importance of UPS in the genesis and progression of ACC and resistance to conventional therapies.

## Supplementary Material

Supplementary Table S1 High-throughput drug screens in ACC cell lines

Supplementary Table S2 Screening for drugs synergistic with TAK-243

Supplementary Table S3 Clinical information for ACC patient derived organoids (PODs)

Supplementary Figure S1 Bortezomib concentration-response curve. Relationship between TAK243 activity and MDR1 and SLFN11 expression.

Supplementary Figure S2 Additive effect of TAK-243 on drugs commonly used to treat ACC.

Supplementary Figure S3 Synergistic effect of TAK-243 on Navitoclax. Analysis of synergistic effects using the MTT assay.

Supplementary Figure S4 Immunostaining of CU-ACC1 xenograft tissue. Body weight changes in mice.

Supplementary Figure S5 Expression of ABCB1/SLFN11 and BCL2/BAX in clinical cancer samples.

Supplementary Figure S6 High UBA1 expression, and hyperactivation of the E1 and E2 enzymes of the ubiquitin pathways in ACC.
