# Supplementary material for: Activity of the Ubiquitin-activating Enzyme Inhibitor TAK-243 in Adrenocortical Carcinoma Cell Lines, Patient-derived Organoids, and Murine Xenografts
Source: Cancer Res Commun. 2024 Mar 19;4(3):834–48. doi: 10.1158/2767-9764.CRC-24-0085 (PMC10949913; doi:10.1158/2767-9764.CRC-24-0085)
Supplement: Supplementary Figure S1 — Bortezomib concentration-response curve. Relationship between TAK243 activity and MDR1 and SLFN11 expression. [file crc-24-0085-s04.pdf]

Supplementary Figure S1

A

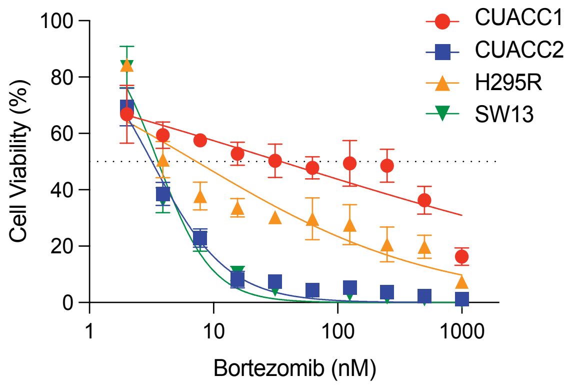

Cell line	CUACC1	CUACC2	H295R	SW13
IC50 (nM)	35.0	3.2	7.3	3.6

B

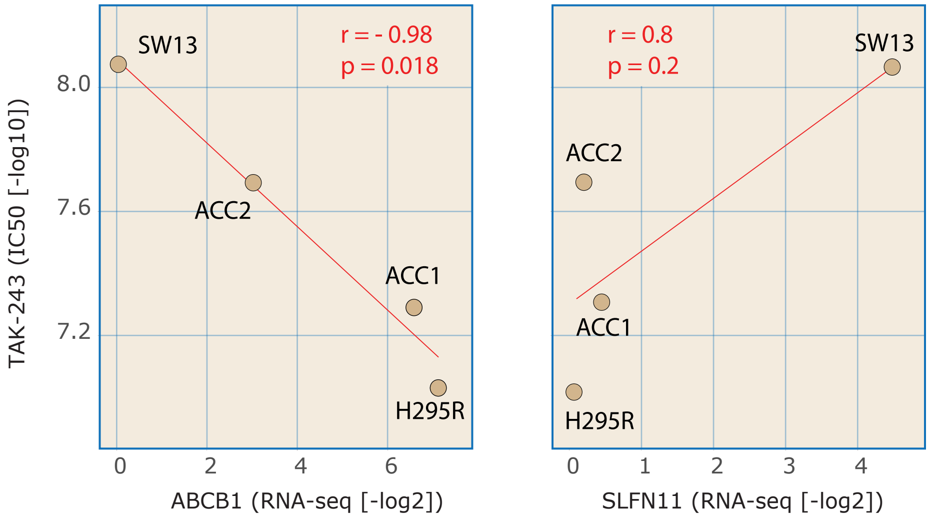

Supplementary Figure S1. A. Concentration-response curves and IC50 values for Bortezomib in ACC cell lines and SW-13. **B.** Activity of TAK-243 is negatively correlated with MDR1 (encoded by *ABCB1*) expression (left) and ACC cell lines have low SLFN11 expression (right) measured by RNA-seq.
