# Supplementary material for: Activity of the Ubiquitin-activating Enzyme Inhibitor TAK-243 in Adrenocortical Carcinoma Cell Lines, Patient-derived Organoids, and Murine Xenografts
Source: Cancer Res Commun. 2024 Mar 19;4(3):834–48. doi: 10.1158/2767-9764.CRC-24-0085 (PMC10949913; doi:10.1158/2767-9764.CRC-24-0085)
Supplement: Supplementary Figure S2 — Additive effect of TAK-243 on drugs commonly used to treat ACC. [file crc-24-0085-s05.pdf]

Supplementary Figure S2

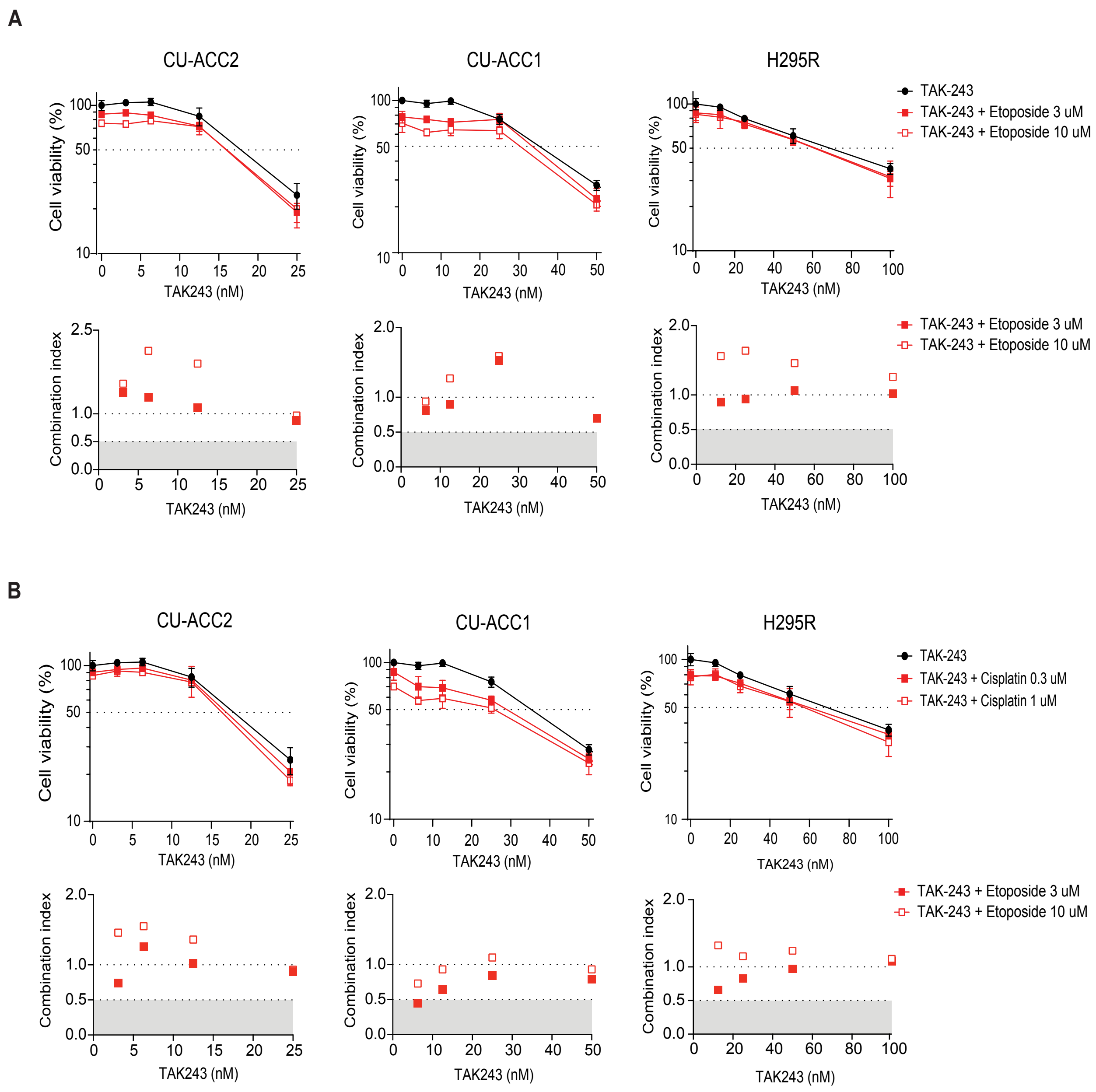

Supplementary Figure S2. Additive effect of TAK-243 on two drugs commonly used to treat ACC. The indicated cell lines were treated with the indicated concentrations of TAK-243 without or with etoposide (**A**) or cisplatin (**B**) for 72 h. Cell viability was evaluated by CellTiter Glo. Error bars represent standard deviations in the triplicate. Combination indexes are plotted for each condition.
