# Supplementary material for: Activity of the Ubiquitin-activating Enzyme Inhibitor TAK-243 in Adrenocortical Carcinoma Cell Lines, Patient-derived Organoids, and Murine Xenografts
Source: Cancer Res Commun. 2024 Mar 19;4(3):834–48. doi: 10.1158/2767-9764.CRC-24-0085 (PMC10949913; doi:10.1158/2767-9764.CRC-24-0085)
Supplement: Supplementary Figure S3 — Synergistic effect of TAK-243 on Navitoclax. Analysis of synergistic effects using the MTT assay. [file crc-24-0085-s06.pdf]

Supplementary Figure S3

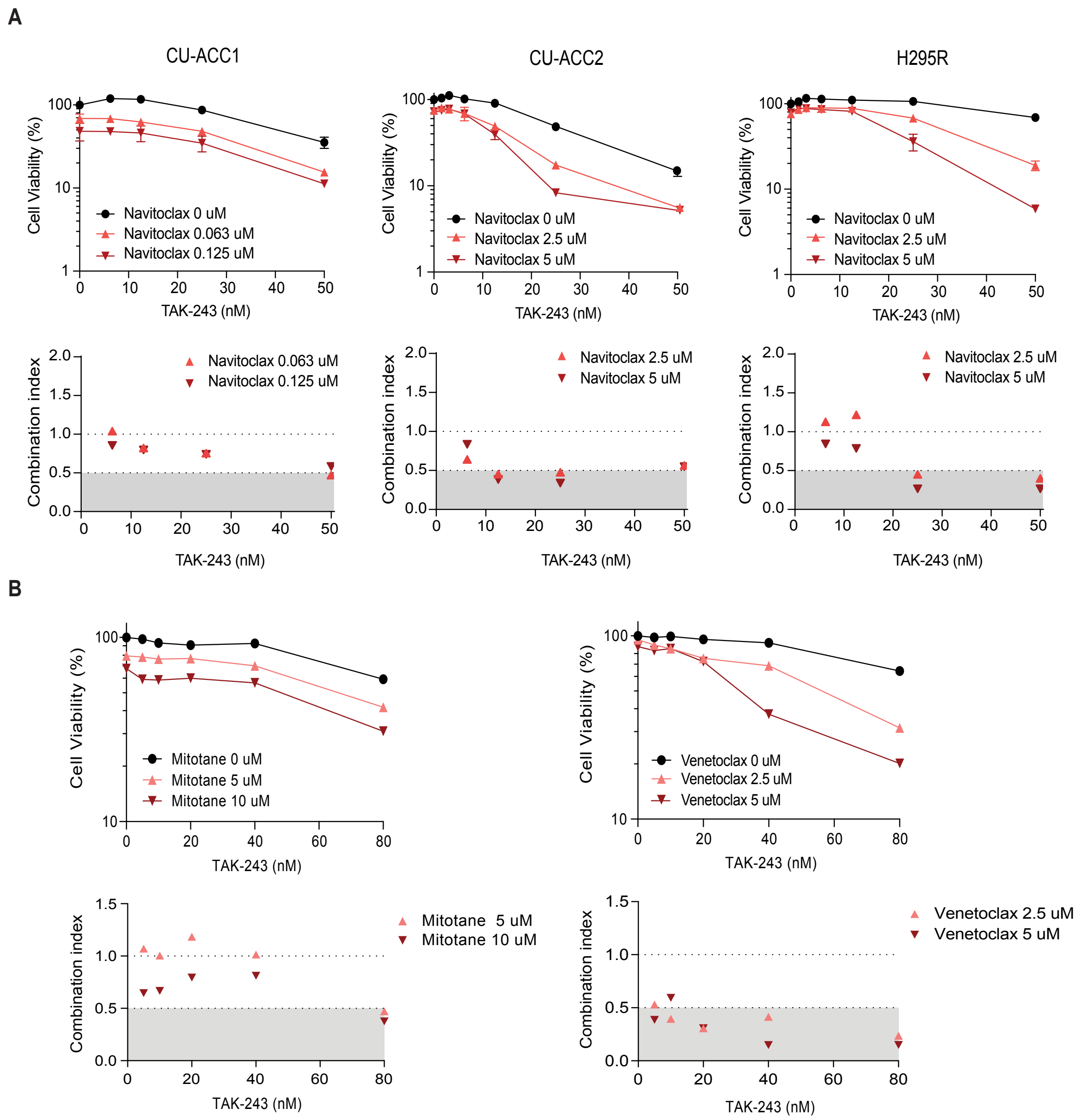


**Supplementary Figure S3. A.** Synergistic effect of TAK-243 on Navitoclax**.** The indicated cell lines were treated with the indicated concentrations of TAK-243 without or with Navitoclax for 72 h. Cell viability was evaluated by CellTiter Glo. Error bars represent stand deviations in the triplicate. Combination indexes are plotted for each condition. **B.** Analysis of synergistic effects using the MTT assay. Left: H295R cells were treated with the indicated concentrations of TAK-243 without or with Mitotane for 72 hours. Cell viability was determined using the MTT assay, and each point on the concentration-response curve represents the mean of duplicate measurements. Right: H295R cells were treated with TAK-243 without or with Venetoclax for 72 hours.
