# Supplementary material for: Activity of the Ubiquitin-activating Enzyme Inhibitor TAK-243 in Adrenocortical Carcinoma Cell Lines, Patient-derived Organoids, and Murine Xenografts
Source: Cancer Res Commun. 2024 Mar 19;4(3):834–48. doi: 10.1158/2767-9764.CRC-24-0085 (PMC10949913; doi:10.1158/2767-9764.CRC-24-0085)
Supplement: Supplementary Figure S4 — Immunostaining of CU-ACC1 xenograft tissue. Body weight changes in mice. [file crc-24-0085-s07.pdf]

Supplementary Figure S4

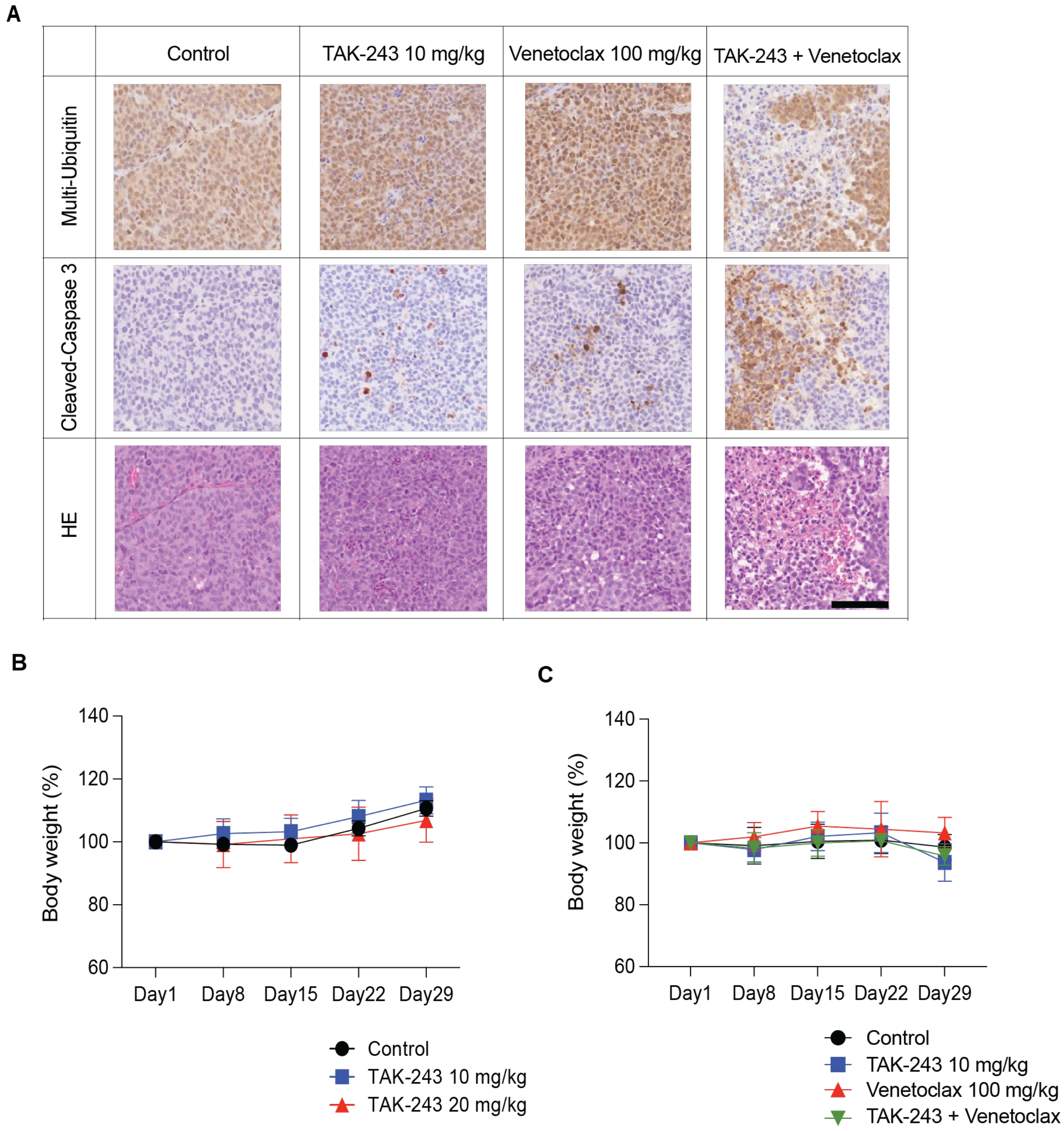

Supplementary Figure S4. Weight change of mice in each treatment group. **A.** H259R xenograft experiment; **B.** CUACC-1 xenograft experiment. Error bars indicate SEM. C. CU-ACC1 xenograft tissues from each treatment group collected on day 29 (top row: immunostaining with anti-multi-ubiquitin antibody, middle row: immunostaining with anti-cleaved caspase-3 antibody, bottom row: HE staining). Scale bar indicates 100 um.
