# Supplementary material for: Activity of the Ubiquitin-activating Enzyme Inhibitor TAK-243 in Adrenocortical Carcinoma Cell Lines, Patient-derived Organoids, and Murine Xenografts
Source: Cancer Res Commun. 2024 Mar 19;4(3):834–48. doi: 10.1158/2767-9764.CRC-24-0085 (PMC10949913; doi:10.1158/2767-9764.CRC-24-0085)
Supplement: Supplementary Figure S5 — Expression of ABCB1/SLFN11 and BCL2/BAX in clinical cancer samples. [file crc-24-0085-s08.pdf]

Supplementary Figure S5.

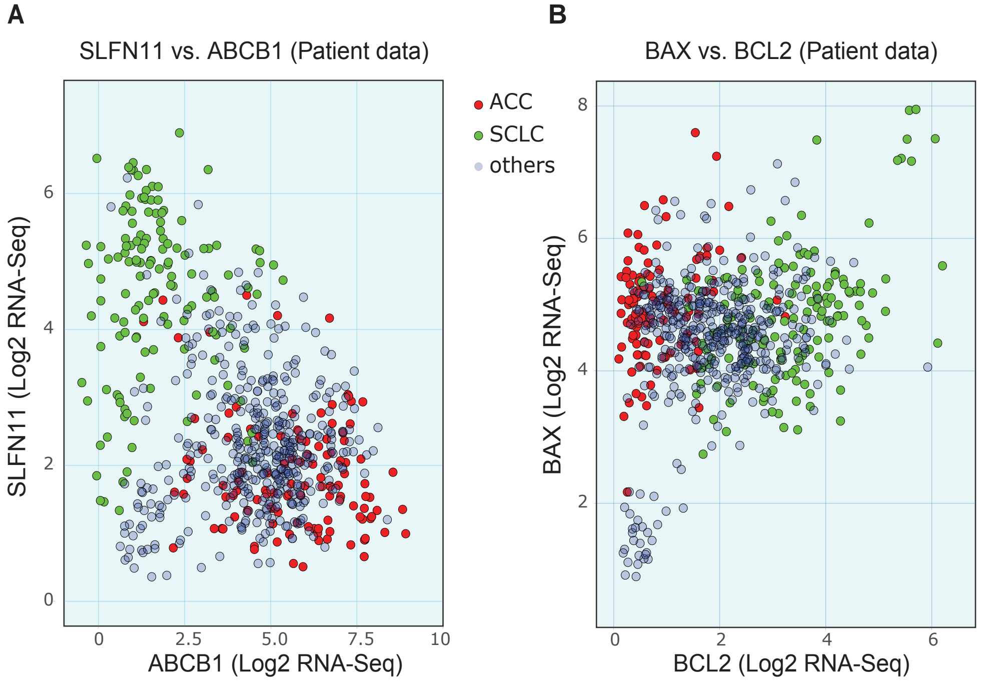

Supplementary Figure S5. Low SLFN11 and BCL2 expression and high ABCB1 and BAX expression in 697 patient samples from TCGA, NCI and other published data. A. ABCB1 (encoding MDR1) vs. SLFN11 expression. B. BCL2 vs. BAX expression. ACC samples (n = 115 samples primarily from TCGA) are colored in red. Small cell lung cancer (SCLC) samples (n = 197 samples from our NCI clinic and published data) are colored in green.
