# Supplementary material for: Activity of the Ubiquitin-activating Enzyme Inhibitor TAK-243 in Adrenocortical Carcinoma Cell Lines, Patient-derived Organoids, and Murine Xenografts
Source: Cancer Res Commun. 2024 Mar 19;4(3):834–48. doi: 10.1158/2767-9764.CRC-24-0085 (PMC10949913; doi:10.1158/2767-9764.CRC-24-0085)
Supplement: Supplementary Figure S6 — High UBA1 expression, and hyperactivation of the E1 and E2 enzymes of the ubiquitin pathways in ACC. [file crc-24-0085-s09.pdf]

Supplementary Figure S6.

A

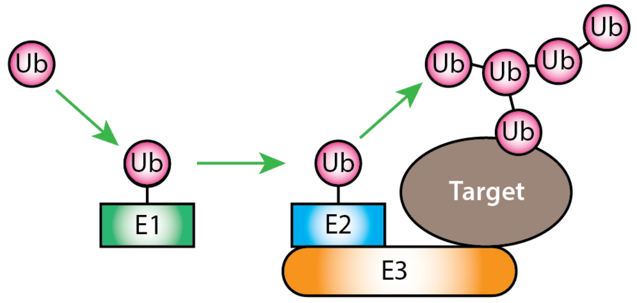

B

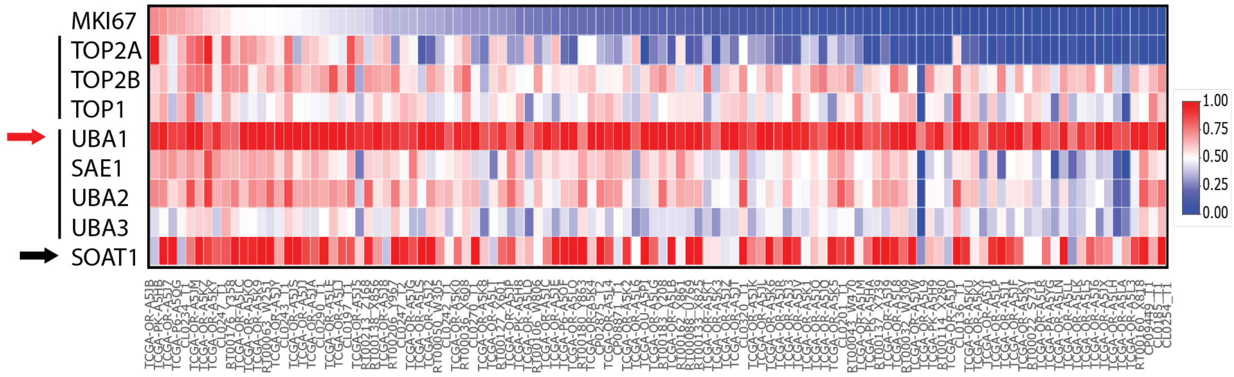

C

E1 gene	Location	Correlation	p-Value
SAE1	19q13.32	0.455	1.76E-07
UBA2	19q12	0.417	2.13E-06
UBA3	3p14.1	0.233	0.0106
UBA1	Xp11.23	0.051	0.581

Supplementary Figure S6. High UBA1 expression (RNA-seq data), and hyperactivation of the E1 and E2 enzymes of the ubiquitin pathways in association with Ki67 expression (poor prognosis) in 120 patient samples from TCGA and the NCI databases. A. Schematic representation of the E1, E2 and E3 enzymes in the ubiquitylation pathway. B. Comparison of UBA1 expression with the expression of the proliferation marker Ki-67 (MKI67) and with topoisomerases II and I, and the 3 other E1 enzymes (SAE1, UBA2 and UBA3) and with the highly expressed Sterol O-Acyltransferase 1 gene (SOAT1) in the CC samples (names listed below each column). C. Correlation between the expression of the E1 enzymes and the proliferation and bad prognosis marker for ACC, MKI67.
